# Ultra high speed SPECT bone imaging enabled by a deep learning enhancement method: a proof of concept

**DOI:** 10.1186/s40658-022-00472-0

**Published:** 2022-06-13

**Authors:** Boyang Pan, Na Qi, Qingyuan Meng, Jiachen Wang, Siyue Peng, Chengxiao Qi, Nan-Jie Gong, Jun Zhao

**Affiliations:** 1 RadioDynamic Healthcare, Shanghai, China; 2 Department of Nuclear Medicine, Shanghai East Hospital, Tongji University School of Medicine, No. 150 Jimo Road, Pudong New District, Shanghai, China; 3 Vector Lab for Intelligent Medical Imaging and Neural Engineering, International Innovation Center of Tsinghua University, No. 602 Tongpu Street, Putuo District, Shanghai, China

**Keywords:** SPECT, Bone, Image quality enhancement, Deep learning

## Abstract

**Background:**

To generate high-quality bone scan SPECT images from only 1/7 scan time SPECT images using deep learning-based enhancement method.

**Materials and methods:**

Normal-dose (925–1110 MBq) clinical technetium 99 m-methyl diphosphonate (99mTc-MDP) SPECT/CT images and corresponding SPECT/CT images with 1/7 scan time from 20 adult patients with bone disease and a phantom were collected to develop a lesion-attention weighted U^2^-Net (Qin et al. in Pattern Recognit 106:107404, 2020), which produces high-quality SPECT images from fast SPECT/CT images. The quality of synthesized SPECT images from different deep learning models was compared using PSNR and SSIM. Clinic evaluation on 5-point Likert scale (5 = excellent) was performed by two experienced nuclear physicians. Average score and Wilcoxon test were constructed to assess the image quality of 1/7 SPECT, DL-enhanced SPECT and the standard SPECT. SUVmax, SUVmean, SSIM and PSNR from each detectable sphere filled with imaging agent were measured and compared for different images.

**Results:**

U^2^-Net-based model reached the best PSNR (40.8) and SSIM (0.788) performance compared with other advanced deep learning methods. The clinic evaluation showed the quality of the synthesized SPECT images is much higher than that of fast SPECT images (*P* < 0.05). Compared to the standard SPECT images, enhanced images exhibited the same general image quality (*P* > 0.999), similar detail of 99mTc-MDP (*P* = 0.125) and the same diagnostic confidence (*P* = 0.1875). 4, 5 and 6 spheres could be distinguished on 1/7 SPECT, DL-enhanced SPECT and the standard SPECT, respectively. The DL-enhanced phantom image outperformed 1/7 SPECT in SUVmax, SUVmean, SSIM and PSNR in quantitative assessment.

**Conclusions:**

Our proposed method can yield significant image quality improvement in the noise level, details of anatomical structure and SUV accuracy, which enabled applications of ultra fast SPECT bone imaging in real clinic settings.

## Background

Single-photon emission computed tomography (SPECT), registered with anatomical imaging CT (together known as SPECT/CT), combines the advantage of molecular-level functional SPECT images and the precise anatomic details of CT images. It has been proved powerful in bone disease diagnosis and is widely used for the detection of bone metastases. Considering the as low as reasonable achievable (ALARA) principle, dose reduction is important. From the imaging technique, reducing radiation dose and shortening scanning time have the same physical essence. The latter also contributes to the patient experience in SPECT/CT examination and reducing the motion artifact. However, short scanning time also means high imaging noise, low image quality and losing diagnostic value. Advanced methods have been proposed to reduce the scanning time or injected dose without degrading the image quality both from the hardware and software sides. In the 1990s, a multi-head gamma camera was introduced to shorten the scan time. In the 2000s, the invention of cadmium zinc telluride (CZT) detector further improved imaging efficiency [1]. Iftikhar Ali et al. [2] adopted ordered-subset expectation maximization with resolution recovery (OSEM-RR) algorithm to keep the image quality for half-time SPECT myocardial perfusion imaging. Aju P. Pazhenkottil [3] proposed a dose-saving algorithm to depress the radiation in SPECT/CT examination.

In recent years, the deep learning method has made massive achievements on multiple imaging tasks like lesion detection [4–6], disease diagnosis [7, 8] and image augmentation [9–11] for different imaging modalities. Some CNN models succeeded to transform low-quality images into high-resolution images with less noise and clear boundaries. For SPECT images, Liu et al. [12] used a 3D coupled U-Net (CU-Net) to suppress imaging noise and artifacts in SPECT myocardial perfusion imaging (MPI). Shiri et al. [13] explored the performance of deep learning method in two time-reduction MPI-SPECT imaging, namely reduction of the acquisition time per projection and reduction of the number of angular projections.

In this work, we collect anonymous clinical SPECT/CT image pairs, enhance the fast acquired image using deep neural network and evaluate the quality of augmented image quantitatively. Compared to previous work, the innovation of this work lies in: (1) we use clinical SPECT image rather than simulated data used in other works; (2) we combine the SPECT image and corresponding CT images which bear richer anatomical information; and (3) we investigate deep learning performance on ultra-high-speed SPECT (1/7 scan time of normal examination), and the noise level is below the standard for making valid clinical diagnosis.

In this paper, we propose a method based on U^2^-Net architecture but can integrate multi-scale and multi-modality features from both low-dose SPECT and corresponding CT images. We incorporated a lesion attention loss function to enhance the sensitivity of our model to reconstruct lesion regions with more accurate SUV measures. Compared to the state-of-the-art methods, our proposed method achieves the best image quality with the highest peak signal-to-noise ratio (PSNR) and structural similarity (SSIM). Furthermore, we evaluate our proposed method on real clinical data and phantom data. It is demonstrated that synthesized SPECT image from fast SPECT with only 1/7 of standard scan time is reliable and provides high agreement and similar diagnostic confidence as standard SPECT imaging in clinical routine, which has great value in real clinical applications.

## Material and methods

### Subjects and image acquisition

**Fig. 1:**
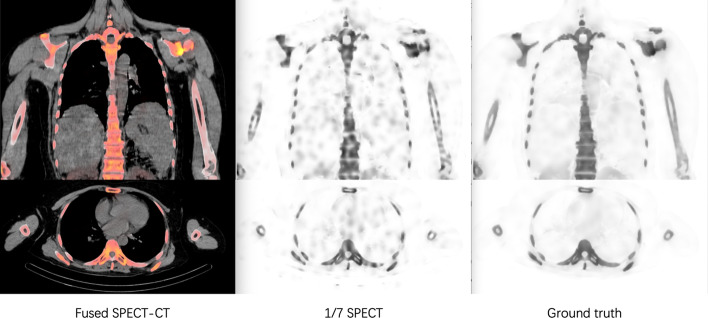
One example of training dataset. The left images are coronal and axial views of fused SPECT and CT images. The middle images are coronal and axial views of 1/7 SPECT. The right images are coronal and axial views of the standard SPECT

Patients received systemic bone imaging after the injection of 25–30 mCi (925–1110 MBq) technetium 99 m-methyl diphosphonate (99mTc-MDP) at Shanghai East Hospital. This study was approved by the Institutional Review Board, and all patients signed informed consent before the examination. The SPECT/CT data were collected using Siemens Symbia Intevo T16 with two continuous protocols: one standard scan with 20 s per projection (referred as the standard SPECT) and one fast scan with 3 s per projection (referred as the 1/7 SPECT). Sixty projections were performed each scan. Projection data were then reconstructed based on CT attenuation map of ordered-subset conjugate gradient (OSCG) algorithm enhanced with 2 subsets and 28 iterations without post-smoothing. Low-dose CT images were collected at 130 kV and 10 valid mAs and reconstructed using a smooth attenuation-correction kernel B31s with 3 mm slice thickness. The resolution of each SPECT images is 256 ×  256, and 200 images were collected each scan. Each SPECT voxel represented a 1.95 mm × 1.95 mm × 1.95 mm space. The resolution of CT images is 512 ×  512, and 131 slices were collected for each patient. Each CT voxel represented a 0.97 mm × 0.97 mm x 3 mm space. All patients were informed to stay still during the examination to keep the quality and correspondence of images. Unmatched 1/7 SPECT and standard SPECT were discarded. Twenty matched groups (11 males, mean age: 56 years, age range: 26–75) of fast and standard SPECT/CT images were collected for further research. Ten subjects were used for training the proposed deep learning model, while the rest 10 subjects were set for testing the synthesized images. One example of training data is shown in Fig. 1.

National Electrical Manufacturers Association (NEMA) International Electrotechnical Commission (IEC) Body Phantom Set was applied as the model, which is a hollow cylinder made of plexiglass with 6 spheres of different diameters (10, 13, 17, 22, 28, 37 mm), and the volume of the hollow cylinder is 9700 ml. The center of the spheres all locates on a circle 5 cm from the center of the cylinder and a plane 70 mm away from the upper surface of the cylinder. SPECT/CT quantitative tomography images with 20 s, 3 s/view and total of 60 views were performed at a specific activity of 12:1 after one hour of instilling. 200 slices of SPECT images and 131 slices of CT images were collected. The first 80 slices of SPECT images and corresponding 53 CT images were inserted to the training dataset, and the rest matched images were treated as testing samples.

### Image preprocess

The simultaneous SPECT and CT acquisition modes facilitate the integration of input data from the two modalities. SPECT image provides diagnostic information at the expense of the anatomic features which can be supplemented by the corresponding CT image. Hence, we propose to combine 1/7 SPECT image and CT image as the input and take the standard SPECT as the network output. To facilitate the combination of SPECT and CT images, each collection of CT images was reshaped into a 200 × 256 × 256 matrix which has the same shape with SPECT. To flatten the difference of voxel values, all the input images and the output were divided by their own average. Then corresponding SPECT and CT slices were concatenate in the first dimension before sending to the proposed U^2^-Net architecture.

The ablation study in the experiment shows the effectiveness of the combination compared with using only 1/7 SPECT as input.

### Residual U-block and U^2^-Net

**Fig. 2:**
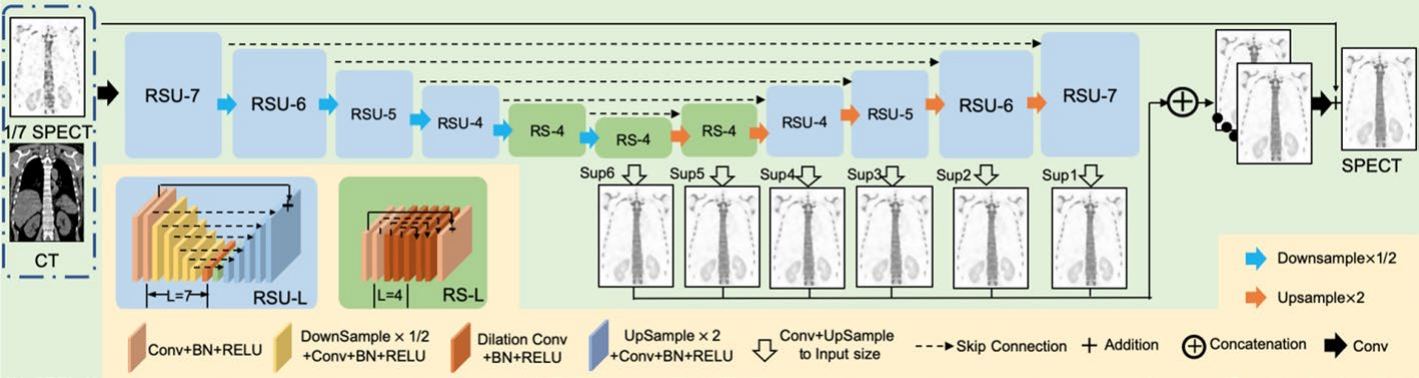
Illusion of proposed U^2^-Net architecture. It consists of 4 residual U-block (RSU) encoders with height of L, 3 bottom residual block (RS) and 4 symmetric RSU decoder. Skip connections are used to save spatial information along matched encoders and decoders. 1/7 SPECT and corresponding CT image are used as the input. The output of the network is images convoluted and upsampled from the second and third RS and follow-up decoders

The 1/7 SPECT varies greatly from the standard SPECT both from the presence of bone structure and voxel value of lesions. So, both local and global contextual features are important for this image synthesis task. U^2^-Net was originally proposed for salient object detection (SOD). The neural network architecture is different from modern CNN designs, such as AlexNet [14], ResNet [15], GoogLeNet [16], DenseNet [17] and VGG [18]. These networks were originally built for image classification tasks which prefer to use small convolutional filters with a size of 1*1 or 3*3 to extract features. U^2^-Net [19] is a simple yet powerful deep network architecture. It contains a novel two-layer nested U-shaped structure [20]. The proposed residual U-block (RSU) consists of a mixture of different-sized receive domains that helps capture contextual information on different scales more efficiently. It also uses pooling operations to increase the overall architecture depth without affecting the computational cost much. There are three major components in RSU which are input convolutional layer, U-Net-like symmetric encoder–decoder structure of ‘L’ height and residual connection to fuse local and multi-scale features using summation. The RSU module with height = 7 is also shown in Fig. 2.

Based on RSU blocks alone, U^2^-Net was developed. It consists of a 6-stage encoder, a 5-stage decoder and a graph fusion module attached to the decoders at different stages. (i) In the encoder stages, RSU-7, RSU-6, RSU-5 and RSU-4 are used, respectively, in which ‘7,’ ‘6,’ ‘5’ and ‘4’ denote the heights (L) of RSU blocks. As the resolution of feature maps in the middle part of U^2^-Net is relatively low, further downsampling of these feature maps would cause loss of useful context. Hence, we use dilated convolution to replace the pooling and upsampling operations, and this kind of special block is referred to as ‘RS-L’ which is also shown in Fig. 2 with height = 4. (ii) In the decoder stages, it has similar structures to their symmetrical encoder stages starting from RS-4. There are 5 stages in total in which each decoder stage takes the concatenation of the upsampled feature maps from its previous stage and those from its symmetrical encoder stage as the input as shown in Fig. 2. (iii) The last graph fusion module is used for generating final synthesized SPECT images. First, this U^2^-Net generates six side output synthesized SPECT images Sup(6), Sup(5), Sup(4), Sup(3), Sup(2), Sup(1), which are upsampled to have the same size as input 1/7 SPECT image. Then, these outputs are fused with a concatenation operation and input to a 1*1 convolution layer followed by a long skip connection with 1/7 SPECT to generate the final synthesized SPECT image.

### Lesion attention loss and deep supervision

To ensure the accuracy of the synthesized image value and distinguishability of the structure and important ROIs, we adapt a combination loss function of the structural similarity index (SSIM) loss and the L1 loss. The total loss for each output SPECT at different decoder stages is

$$L = L_{1} + \alpha L_{{{\text{ssim}}}}$$

where $\alpha$ is a fixed weight ($\alpha$ = 0.5) that balances the SSIM loss and L1 loss.

To further improve the performance on lesion regions, we add lesion attention masks to emphasize the loss in these areas. The lesion masks were contoured on standard SPECT by physicians. So, the improved lesion attention loss is defined as

$$\ell = L + \beta L*M$$

where $\beta$ is a fixed weight ($\beta$ = 100) that balances the lesion region loss and whole image loss. *M* is the lesion mask.

We also use deep supervision strategy in the training process to speed up the training process and acquire stable media layers. The total loss for training the U^2^-Net is defined as

$${\mathcal{L}}_{{{\text{total}}}} = \mathop \sum \limits_{i = 1}^{N} w_{{{\text{side}}}}^{i} \ell_{{{\text{side}}}}^{i} + w_{{{\text{final}}}} \ell_{{{\text{final}}}}$$

where $\ell_{{{\text{side}}}}^{i}$ (*N* = 6, as Sup1, Sup2, Sup3, …, Sup6 in Fig. 2) is the loss of the side output and $\ell_{{{\text{final}}}}$ is the loss of final output of the network. $w_{{{\text{side}}}}^{i}$ and $w_{{{\text{final}}}}$ control the weights of each component in the total loss. In the testing process, final output is the only part for synthesizing SPECT images.

### Implementation details

The proposed method is implemented using PyTorch 1.6.0 and trained on four NVIDIA GEFORCE 3090 (24 GB). The network is trained for 100 epochs, and the batch size is set to 4 by using axial slice as inference plain. VGG net is used as the discriminator. Adam optimizer is used with the learning rate of 0.0002 for both the generator and discriminator and divided by 10 after 80 epochs.

### Quantitative assessment

To quantitatively evaluate the performance of synthesized images, PSNR and SSIM are used as evaluation metrics. PSNR for synthesized image is defined as

$${\text{PSNR}} = 10 \cdot \log_{10} \left( {\frac{{{\text{MAX}}_{{{\text{gt}}}}^{2} }}{{{\text{MSE}}}}} \right)$$

where ${\text{MAX}}_{{{\text{gt}}}}$ is the maximum pixel value of ground truth standard SPECT. MSE is the mean square error of synthesized images compared to the standard SPECT.

SSIM for synthesized image is defined as

$${\text{SSIM}}\left( {x,y} \right) = \frac{{\left( {2\mu_{x} \mu_{y} + c_{1} } \right)\left( {2\sigma_{xy} + c_{2} } \right)}}{{\left( {\mu_{x}^{2} + \mu_{y}^{2} + c_{1} } \right)\left( {\sigma_{x}^{2} + \sigma_{y}^{2} + c_{2} } \right)}}$$

where $\mu_{x}$ and $\sigma_{x}^{2}$ are average value and variance of input synthesized image. $\mu_{y}$ and $\sigma_{y}^{2}$ are the average value and variance of input standard SPECT. $\sigma_{xy}$ is covariance of two images. $c_{1}$ and $c_{2}$ are small constants. SSIM is calculated using scikit-image package.

### Clinical assessment

Two readers independently grade 1/7 SPECT, synthesized SPECT by proposed method and standard SPECT in terms of general image quality, detail of 99mTc-MDP distribution, presence of artifacts and general diagnostic confidence using 5-point Likert scale (1 for unacceptable image quality; 2 for suboptimal image quality; 3 for acceptable image quality; 4 for good image quality; and 5 for excellent image quality). Readers are blinded to meta-information of compared images.

Average scores of each kind of image are compared. Paired t test is used to identify significant differences between each criterion.

### Phantom study

Half phantom images are used to training the model. The rest half including the center position of the phantom are used for testing. The images of the sphere centers are used to distinguish different spheres. SUVmax and SUVmean are measured and compared for each recognizable sphere. SUV is defined as

$${\text{SUV}} = {\text{pixel}}\;{\text{value}}*{\text{weight}}\;{\text{in}}\;{\text{grams}}/{\text{total}}\;{\text{dose}}\;{\text{in}}\;{\text{MBq}}{.}$$

PSNR and SSIM are calculated for 1/7 SPECT and generated SPECT.

## Results

### Comparison with other networks

The proposed U^2^-Net model is compared with four widely used deep learning architectures, i.e., EDSR [21], RCAN [22] and ESRGAN [23], respectively.

**Fig. 3:**
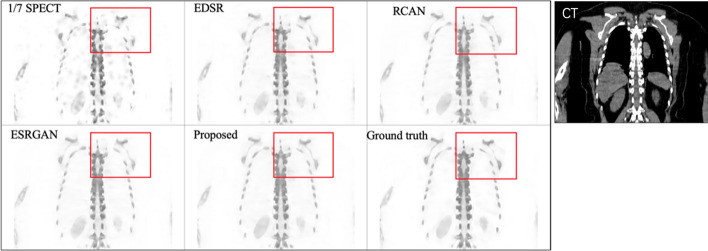
Visual results of different methods on synthesizing standard SPECT from 1/7 SPECT. Please zoom in for a better comparison. The results are images from the same case using 1/7 scan time constructed with the OSCG algorithm, enhanced by EDSR network, RCAN network, ESRGAN network, proposed U^2^-Net, respectively, and the standard SPECT image

**Fig. 4:**
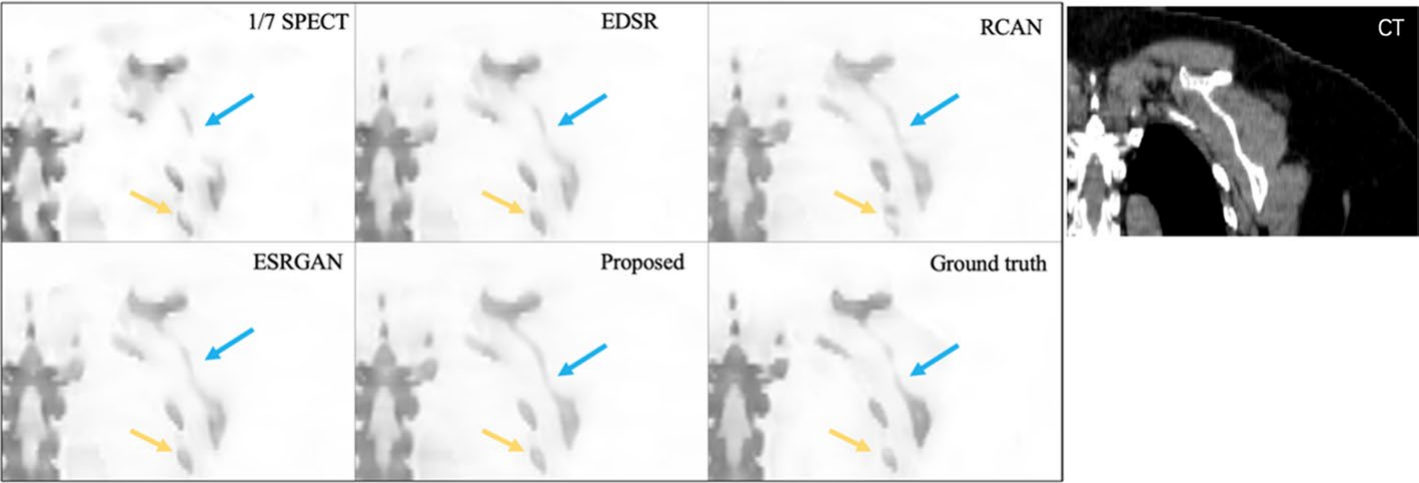
Zoomed visual results of different methods on synthesizing standard SPECT from 1/7 SPECT. The results are zoomed images from the same case using 1/7 scan time constructed with the OSCG algorithm, enhanced by EDSR network, RCAN network, ESRGAN network, proposed U^2^-Net, respectively, and the standard SPECT image

**Fig. 5:**
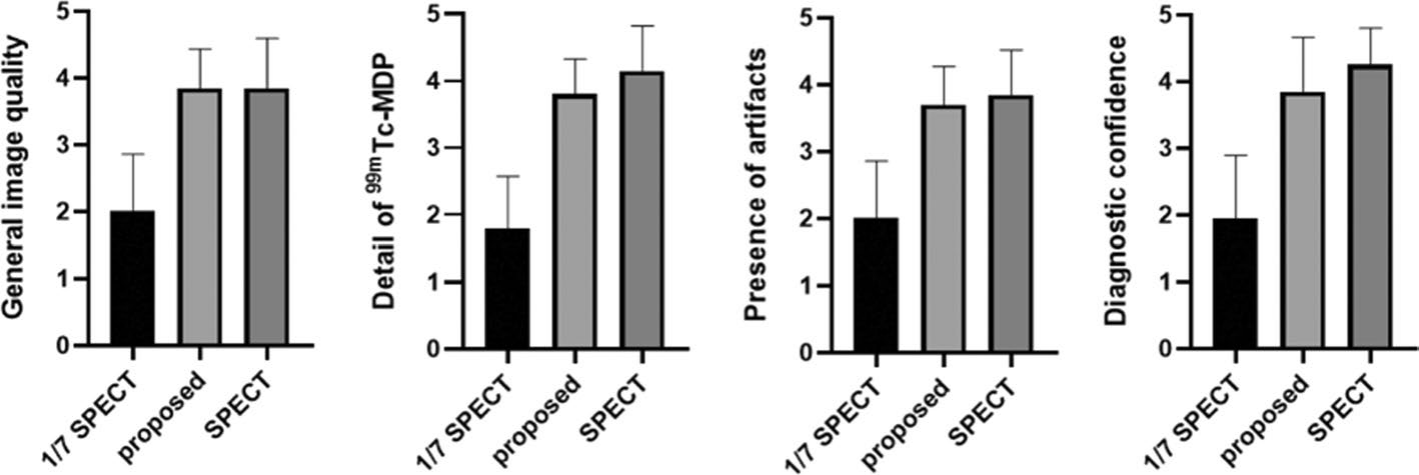
An axial view of image difference between different methods and the standard SPECT. **a**–**f** The original image of 1/7 SPECT, EDSR, ESRGAN, RCAN, proposed method and the standard SPECT in the grayscale of minimum = 0, maximum = 120,000. **g**–**k** The difference between the standard and corresponding image (standard–corresponding image, minimum =  − 20,000, maximum = 20,000)

The visualization results for one testing case for different methods are shown in Fig. 3. A zoomed-in region is also shown in Fig. 4 for better comparison. Figure 5 provides an axial view of image difference between different methods and the standard SPECT.

The quantitative results are presented in Table 1. In general, our result reaches the best performance based on both qualitative and quantitative evaluation. The noise in the chest region as shown in the 1/7 SPECT image has been successfully removed in our proposed result. This is the effect of using U^2^-Net as the neural network architecture since it provides abundant contextual information from different scales for improving the anatomical structural details. This is also the effect of adding the CT image as the input of the network due to the clear anatomy feature provided by CT images which results in clear boundaries in the synthesized standard SPECT image. Our proposed method also increases the sharpness with consistent details and achieves the highest PSNR and SSIM score at the same time (PSNR = 40.8, SSIM = 0.788).

**Table 1:** Average SSIM and PSNR comparison for different methods

	1/7 SPECT	EDSR	RCAN	ESRGAN	Proposed
SSIM	0.765	0.778	0.781	0.772	0.788
PSNR	37.7	38.6	40.5	40.1	40.8

### Clinic evaluation

**Fig. 6:**
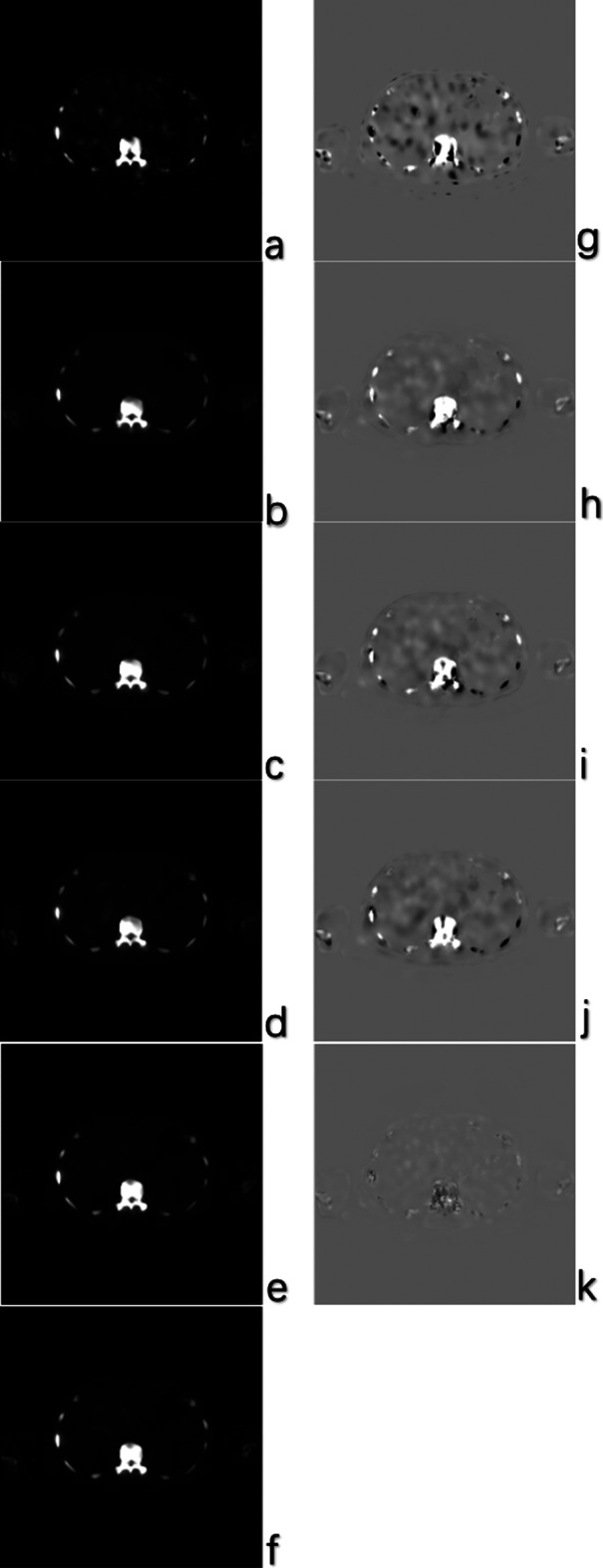
Clinical assessment results of image quality for 1/7 SPECT, proposed method and the standard SPECT. The average scores for the 1/7 SPECT, proposed method and the standard SPECT are 2, 3.85 and 3.85 in terms of general image quality, 1.8,3.8 and 4.15 for details of contrast, 2, 3.75 and 3.9 for presence of artifacts and 1.95, 3.85 and 4.15 for diagnostic confidence. 1/7 SPECT is significantly different from SPECT image enhanced by proposed method under Wilcoxon test (*P* < 0.05) for all evaluation metrics. Compared to the standard SPECT, proposed method achieved the same general image quality (*P* > 0.999), similar detail of 99mTc-MDP (*P* = 0.125), presence of artifacts (*P* = 0.531) and the diagnostic confidence (*P* = 0.1875)

The assessment results are shown in Fig. 6. The average scores for the 1/7 SPECT, proposed method and the standard SPECT are 2, 3.85 and 3.85 in terms of general image quality, 1.8,3.8 and 4.15 for details of contrast, 2, 3.75 and 3.9 for presence of artifacts and 1.95, 3.85 and 4.15 for diagnostic confidence. We can see the average grades of the proposed method are much better than those of 1/7 SPECT. Significant difference was found using Wilcoxon test (*P* < 0.05) for all evaluation metrics. Compared to the standard SPECT, our method achieved the same general image quality (*P* > 0.999), similar detail of 99mTc-MDP (*P* = 0.0510), presence of artifacts (*P* = 0.3434) and the diagnostic confidence (*P* = 0.1265).

### Phantom study

**Fig. 7:**
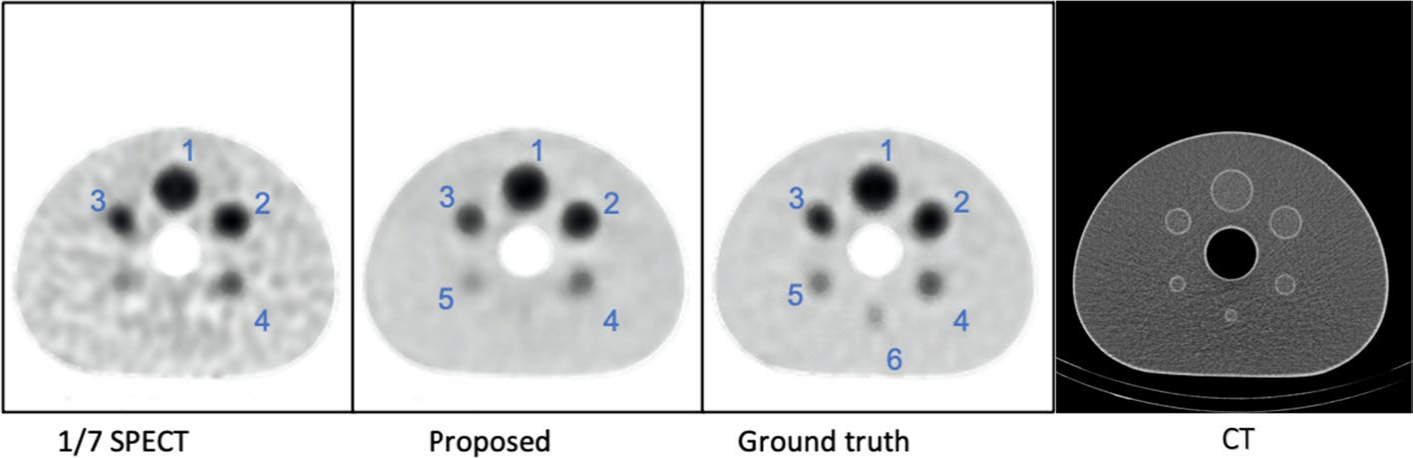
Visualization results of phantom images from different methods. On 1/7 SPECT, only 4 spheres can be identified that exhibited evident contrast against background signals. On the generated image and the ground truth image, 5 and 6 spheres can be distinguished separately

The phantom images acquired from 1/7 of standard scan time are also enhanced using our method. The images of the center part of the phantom are shown in Fig. 7. On 1/7 SPECT, we can only identify 4 spheres that exhibited evident contrast against background signals. On the generated image and the ground truth image, we can distinguish 5 and 6 spheres separately. All these spheres are numbered in the figure for further quantitative evaluation.

SUVmax and SUVmean are calculated for spheres 1 to 4. The quantitative results are shown in Table 2. SUVmax and SUVmean of each sphere in enhanced image are much closer to the ground truth image compared with the 1/7 SPECT image.

**Table 2:** Quantitative results for different spheres

	SUVmax			SUVmean		
	1/7 SPECT	Proposed	Ground truth	1/7 SPECT	Proposed	Ground truth
Sphere 1	12.314	13.591	13.756	9.529	10.216	10.379
Sphere 2	13.176	12.4	12.231	8.405	8.095	7.923
Sphere 3	9.733	9.385	9.292	7.366	7.275	7.284
Sphere 4	4.897	4.62	4.735	4.093	3.869	3.885

SSIM and PSNR results for 1/7 SPECT and enhanced image using the standard SPECT as reference are shown in Table 3. Proposed method performed better than 1/7 SPECT on both criteria.

**Table 3:** Average SSIM and PSNR comparison for 1/7 SPECT and enhanced image

	1/7 SPECT	Proposed
SSIM	0.87	0.94
PSNR	32.12	33.54

### Ablation study

**Fig. 8:**
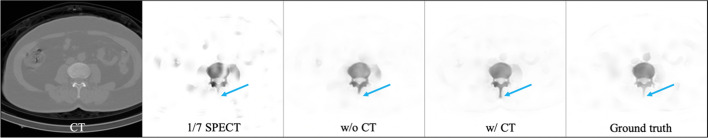
Visual results of w/ and w/o CT as input of the proposed model. Enhanced image with combined CT input preserved more structure than those without CT input and 1/7 SPECT

**Fig. 9:**
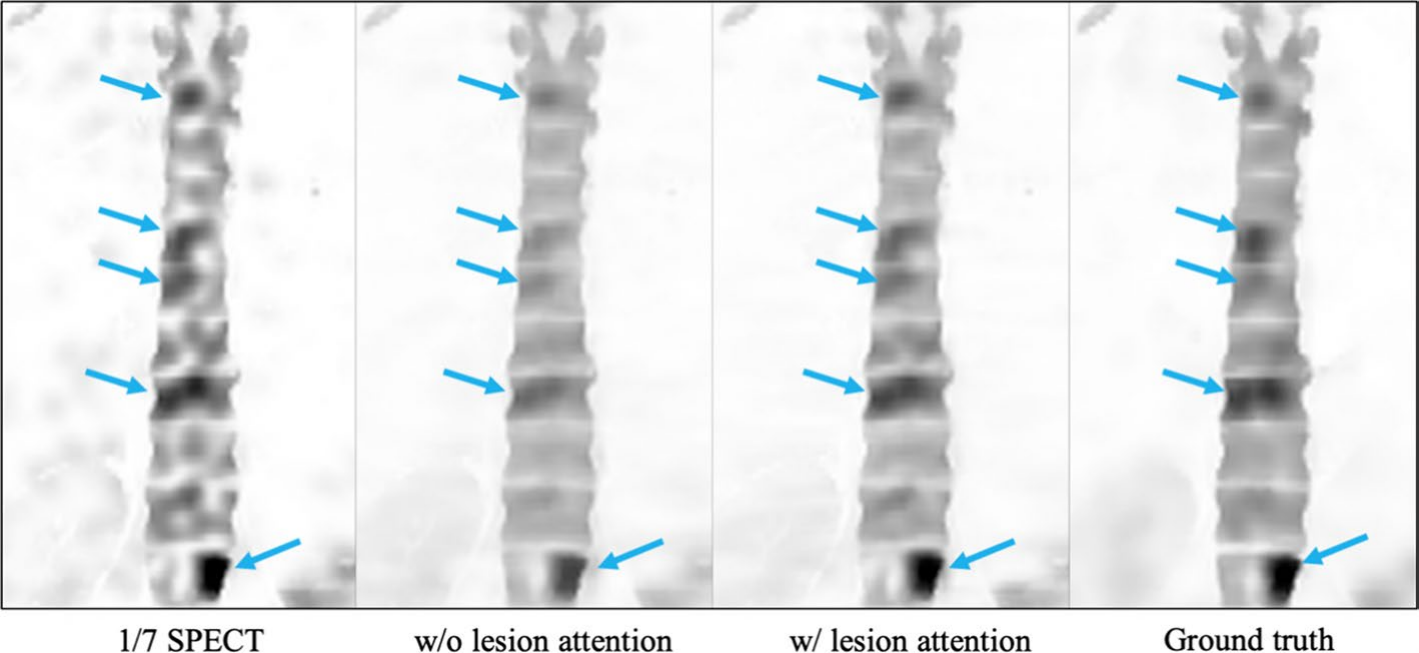
Visual results of w/ and w/o lesion attention as input of the proposed model. Enhanced image with lesion attention loss holds clearer SUV variation within tissues than those without lesion attention

To verify the effectiveness of using both 1/7 SPECT and CT image as input that boosts the image quality, ablation studies are conducted on w/ or w/o CT, while another implementation set remains the same. The visual results are shown in Fig. 8, in which we can observe that the bone structure pointed by the blue arrow has been recovered in the result with both 1/7 SPECT and CT as input, while the result w/o CT as input fails to recover this bone structure due to the missing information in the original 1/7 SPECT image. Ablation study of w/o and w/ lesion attention loss is also shown in Fig. 9. We can see that the synthesized SPECT using lesion attention has more accurate SUV values and better contrast compared to w/o lesion attention as pointed by the blue arrow.

## Discussion

The deep learning-based imaging enhancement method has been applied to many modalities like low-dose PET/MRI [24], compressive sensing MRI [9] and low-dose CT [25]. For SPECT images, Olia et al. [26] implemented a generative adversarial network to predict non-gated normal-dose SPECT-MPI images in the projection domain at different reduced dose levels. Song et al. [27] investigated a 3D residual convolutional neural network (CNN) model to predict standard-dose images from 1/4-dose gated SPECT-MPI images. However, no research has employed an artificial intelligence algorithm on SPECT bone image augmentation to the best of our knowledge. Compared with previous SPECT study, Olia et al. and Song et al. use simulated low-dose SPECT reconstructed from partial list mode data, and our work uses continuous scan to acquire data pairs which is closer to clinical settings. Besides, we explored a more complicated network architecture which could extract smaller features.

This study evaluated 1/7 SPECT/CT image augmentation using the proposed deep learning method on a clinical set. The results showed that an undiagnosable SPECT image could be enhanced by deep learning method to be comparable with the standard SPECT in terms of visual effect. Quantitative results showed that PSNR and SSIM of synthesized SPECT images were much better than the original 1/7 SPECT and the proposed method reached the best performance compared with other advanced deep learning methods.

Organ movements during acquisition series may lead to non-perfect alignment of CT to SPECT and fast scan to the standard scan. Our result shows this phenomenon is not severe in organs visible in SPECT images like kidney. Our result shows the boundary of these soft tissues remains clear. The main problem of soft tissue quality is still noise level.

Clinic assessment exhibits that the clinical value of synthesized images is much better than this of low-dose SPECT images and comparable to the standard SPECT images. The average score for deep learning enhanced image is obviously higher 3 and close to 4 in 5-point Likert scale, which proved the image quality meets the standard for clinic use. Dietze et al. [28] compared different methods for SPECT image reconstruction including filtered back projection (FBP), clinical used method, convolution neural network (CNN)-based method and Monte Carlo-based method. They concluded that images generated by CNN-based method reached similar image quality as those reconstructed by the Monte Carlo method but spent far less time. This result also demonstrated the potential of CNN-based imaging enhancement method in clinical use.

Compared to other widely used network architectures, our proposed U^2^-Net reached the best PSNR and SSIM performance. U^2^-Net has been verified useful in image segmentation task [19], but the augmentation task is quart different in predicting the exact value of each pixel. Our visual results (Figs. 3, 4) proved U^2^-Net-based method has the best capability to handle small structures which could be discontinuous or invisible on 1/7 SPECT images. Since no bone scan has been enhanced by deep learning method, we compared our results with other SPECT study. Li et al. [29] compared SSIM and PSNR of DL-enhanced phantom images on different dose levels. They received 0.9470 SSIM of simple Jaszczak phantom from 60-view projection data and 0.9117 from 30-view projection data. It is higher than our clinical result and comparable to our phantom result. High noise level in background is the main reason accounting for the relatively low SSIM result. If we set the background all to 0, we got SSIM of about 0.87 for 1/7 SPECT and about 0.96 for proposed method which is on the same level as Li’s result.

The ablation study showed the effectiveness of combining both SPECT and corresponding CT as the input, which makes it possible to recover the bone structures that were missing on 1/7 SPECT. Associating a functional image and the matching structural image as the input of the neural network for image augmentation task has been proved effective in low-dose PET image recovery task [24]. Our result showed the possibility of structure recovery in fast SPECT that not presented on the standard SPECT. This means the input CT image may not only provide the structure information but also contribute to the pixel value which make the result misleading. One solution to this issue could be use the binarized gradient map of CT image which only provide structure boundaries. Increasing the training dataset may also help to solve this problem by generating a more precise model.

The ablation study also proved the usefulness of adding lesion region loss in the training process, which retrieved the contrast within structures much better than those without lesion region loss. The SUV value in lesion area is more important in the clinical criteria. Ly et al. [30] evaluated the SUV of low-dose PET, the standard PET and PET synthesized using deep learning method. The simulated image has equal SUV performance as the low-dose image, but far less than full-dose PET image. Our results exhibit that the SUV distribution with lesion attention is much more precise than those without lesion attention, which means the strategy also contributed to the accuracy of SUV.

Our experiment proved the feasibility of greatly accelerating SPECT imaging without sacrificing image quality using deep learning method. The fast SPECT imaging technique ameliorates patient experience and reduces motion artifacts. Methods have been proposed to speed up SPECT imaging [1, 2, 12, 13]. However, traditional methods like using advanced detectors or using iterative algorithms could only increase the imaging efficiency by a factor of two, and our methods could apply to ultra-low-dose SPECT imaging with only 1/7 scan time. Compared to other DL-based methods applying to MPI-SPECT, our method improved the imaging quality of bone SPECT. We also made it possible to make the image quality of synthesized SPECT comparable to that of the standard acquisitions.

In this work, we also applied our method to the phantom data. Chrysostomou et al. [31] used deep learning method to reconstruct low-projection SPECT imaging. They proved SSIM, MSE and PCC of enhanced phantom images were better than that of simulated low-projection SPECT images. In our study, we scan the real phantom and measured SSIM, PSNR, SUVmax and SUVmean and reach the same results as the previous work.

Our method could also enhance the image quality of low-dose SPECT images for both shortening the scan time and reducing the amounts of injected radionuclides, resulting in the reduction of received signals for SPECT scanners, which is the direct factor affecting SPECT image quality. Ramon et al. [32] evaluated the diagnostic accuracy of CNN-enhanced low-dose MPI-SPECT image and draw a conclusion that DL denoising can achieve additional dose reduction without sacrificing the diagnostic accuracy in SPECT-MPI compared with iteration algorithms.

One limitation of our study is that our method is tested on a single SPECT/CT system, single contrast agent and the same imaging reconstruction method. Further, we did not study the impact on image quality in special patient groups such as obese patients, young or old patients and patients with other metabolic diseases. Nevertheless, this is the first study to evaluate the performance of deep learning method on fast bone SPECT using clinical data. Further study in a larger population is required to explore the optimum weights for different parts of the total loss and cut off the extra channels in the network to speed up the calculation. We believe this method could also be applied to greatly reducing injection dose if further validated.

## Conclusion

In this paper, we focus on the synthesis of standard SPECT from a ultra fast SPECT scan with only 1/7 scan time. We propose to apply a novel U^2^-Net-based framework that aggregates features from both fast SPECT scan and corresponding CT image as well as extract both local and global information from multi-scale features. We demonstrated the proposed method  was able to achieve an acquisition time reduction by 7 times. The comprehensive results showed the proposed method can yield significant image quality improvement both in the noise level, anatomical structure clearness and SUV accuracy which enables application in real clinical settings.

## Data Availability

The datasets used and/or analyzed during the current study are available from the corresponding author on reasonable request.

## Abbreviations

ALARA: As low as reasonable achievable
CNN: Convolution neural network
CU-Net: Coupled U-Net
CZT: Cadmium zinc telluride
DL: Deep learning
FBP: Filtered back projection
MPI: Myocardial perfusion imaging
NEMA: National electrical manufacturers association
OSEM-RR: Ordered-subset expectation maximization with resolution recovery
PSNR: Peak signal-to-noise ratio
RS: Residual block
RSU: Residual U-block
SOD: Salient object detection
SSIM: Structural similarity index
SUV: Standard uptake value
SUVmax: Maximum standardized uptake value
SUVmean: Mean standardized uptake value
99mTc-MDP: Technetium 99 m-methyl diphosphonate
